# Brain Specific RagA Overexpression Triggers Depressive‐Like Behaviors in Mice via Activating ADORA2A Signaling Pathway

**DOI:** 10.1002/advs.202404188

**Published:** 2024-10-07

**Authors:** Jia Zhao, Yilu Sun, Yibin Feng, Jianhui Rong

**Affiliations:** ^1^ School of Chinese Medicine Li Ka Shing Faculty of Medicine The University of Hong Kong 3 Sassoon Road, Pokfulam Hong Kong 999077 P. R. China; ^2^ Department of Chinese Medicine The University of Hong Kong Shenzhen Hospital Shenzhen 518053 P. R. China

**Keywords:** ADORA2A, depression, molecular mechanism, p70S6K, RagA

## Abstract

Neuroinflammation hallmarks the pathology of depression although the etiological complexity has not yet been resolved. Previous studies demonstrate that bacterial lipopolysaccharide induces depressive‐like behaviors by activating RagA‐mTOR‐p70S6K signaling pathway. The current project aims to investigate whether and how brain‐specific RagA overexpression triggers depressive‐like behaviors in mice. Full‐length RagA cDNA is cloned into the mammalian expression vector under the control of brain specific promoter, and subsequently overexpressed in the brain of mouse embryos. Indeed, RagA transgenic mice exhibit depressive‐like behaviors and memory impairments. RNA‐seq profiling of the prefrontal cortex (PFC) transcriptome highlights adenosine A2a receptor (ADORA2A) as a key differentially expressed gene (DEG). Western blotting confirms that ADORA2A and phospho‐p70S6K are markedly elevated in RagA transgenic mice. Behavioral assessments demonstrate that ADORA2A inhibitor istradefylline markedly attenuates depressive‐like behaviors. Further metabolomics reveals that N‐acetylserotonin and several depression‐related metabolites are downregulated while proteomic profiling showed that OLIG1 and other proteins are significantly regulated in RagA transgenic mice. Collectively, RagA overexpression alters the expression patterns of signaling proteins and the metabolism of depression‐associated metabolites. RagA may cause depressive‐like behaviors in mice via activating p70S6K/ADORA2A signaling pathway. Thus, RagA‐p70S6K‐ADORA2A signaling pathway may be a target for the development of new antidepressant therapies.

## Graphical abstract

The overexpression of brain‐specific RagA leads to depressive‐like behaviors, memory impairment, and hippocampal neuron loss in mice. This is accompanied by an upregulation of the adenosine A2a receptor (ADORA2A) and coordinated changes in the levels of depression‐related metabolites, proteins, and lncRNA. The RagA‐ADORA2A pathway is a new target for the development of new antidepressant drugs.

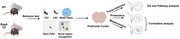

## 1 Introduction

Depression is a prevalent devastating mental disorder and seriously affects 5% of global adult population from the quality of life, the harmony of society, and the economy of the communities.^[^ ^1^ ^]^ The complex etiology of depression possibly involves genetic alterations, endocrinal dysfunctions, mental stress, unhealthy living habits, and comorbid illnesses.^[^ ^2^ ^]^ Pathologically, neuroinflammation and immune dysregulation are recently recognized as the causative factors for the onset and progression of depression.^[^ ^3^ ^]^ Neuroinflammation links chronic stress to the dysregulation of the hypothalamic‐pituitary‐adrenal (HPA) axis, the overproduction of stress hormones and the hyperactivation of the central and peripheral immune cells, ultimately leading to the development of psychological and metabolic disorders.^[^ ^3^, ^4^ ^]^ Interestingly, bacterial lipopolysaccharide (LPS) not only triggers an acute inflammatory response, but also induces depressive‐like behaviors in mice.^[^ ^5^ ^]^ By profiling the transcriptomic response of mouse brain to LPS challenge, we recently demonstrated that LPS activated the mechanistic target of rapamycin (mTOR) pathway by upregulating the expression of the lysosomal guanosine triphosphatase (GTPase) RagA.^[^ ^6^ ^]^ Indeed, bacterial infection activates RagA‐mTOR‐p70S6 kinase (p70S6K) axis to induce depression. Nevertheless, naturally occurring isoflavone puerarin could effectively ameliorate LPS‐induced depressive‐like behaviors via downregulating RagA expression.^[^ ^7^ ^]^ These results suggest that new pharmacological inhibitors of RagA‐mTOR‐p70S6K pathway may help the treatment of depression.

The protein mTOR not only exhibits the activity of an atypical serine/threonine kinase, but also forms two major complexes with distinct proteins in the control of cell growth, carcinogenesis, diabetic progression, aging, psychological status, and memory.^[^ ^8^ ^]^ The mTOR complex 1 (mTORC1) involves mTOR, Raptor, GβL, and DEPTOR for the regulation of cell cycle, macromolecule biosynthesis, metabolism, cell growth, and autophagy.^[^ ^9^ ^]^ On the other hand, the mTOR complex 2 (mTORC2), involves mTOR, Rictor, GβL, Sin1, PRR5/Protor‐1, and DEPTOR for the regulation of cell survival, cytoskeletal dynamics, and metabolism.^[^ ^9^ ^]^ The small Rag GTPases are known to sense the intracellular levels of glucose and amino acids for the mTOR complex 1 (mTORC1) pathway.^[^ ^10^ ^]^ Upon stimulation by amino acids, Rag GTPase isoforms (i.e., RagA, RagB, RagC, RagD) form heterodimers to recruit and activate mTORC1 in lysosomes.^[^ ^11^ ^]^ The activation of mTORC1 subsequently leads to the transduction of cell signals through two key downstream substrates, namely, p70S6K and eukaryotic initiation factor 4E‐binding protein 1 (4EBP1), to regulate cognitive functions and neuropsychiatric conditions.^[^ ^12^ ^]^ However, RagA expression is tightly regulated in mammals while either deletion or knock‐in of RagA causes early death in mice.^[^ ^10^, ^13^ ^]^ Consequently, little is known about the mechanism by which RagA promotes depression.

In this study, therefore, we selectively overexpressed RagA in mouse brains and assessed the behaviors of RagA transgenic mice while wild‐type mice were tested as control. We isolated the total RNAs from both wild‐type and RagA transgenic mice and profiled the transcriptomes using next‐generation RNA sequencing. In parallel, we also analyzed the alterations of metabolites and proteins by metabolomics and proteomics technologies. We further verified the potential targets in RagA transgenic mice, highly differentiated PC12 cell line and hippocampal HT‐22 cell line.

## 2 Results

### 2.1 RagA Transgenic Mice Overexpressed RagA in the Frontal Cortex

**Figure 1:**
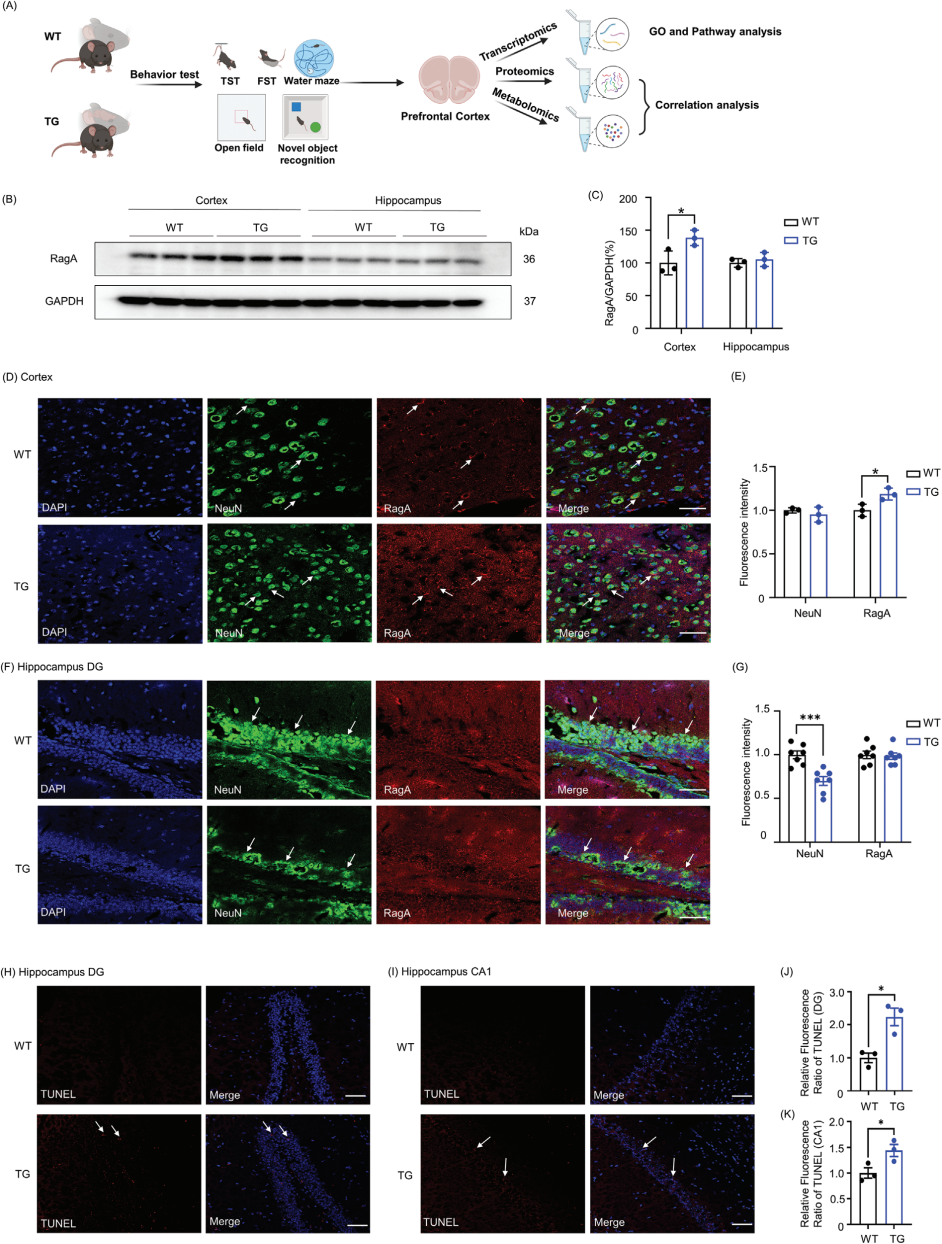
RagA was upregulated in the prefrontal cortex (PFC) of RagA transgenic mice. A) Schematic representation of the animal experiments. B) The representative band of RagA in the PFC and hippocampus of WT mice and RagA transgenic (TG) mice. C) Quantitative analysis of RagA. The western blots in panel B were analyzed using a densitometric method (n = 3). D) Representative images of RagA (red), NeuN (green) and DAPI (blue) immunostaining in the PFC of WT mice and TG mice. Arrows pointed to the colocalization of RagA and NeuN. Scale bar, 50 µm. E) Quantitative analysis of RagA and NeuN. Fluorescence intensity of RagA and NeuN in panel D was measured using a densitometric method (n = 3). F) Representative images of RagA (red), NeuN (green) and DAPI (blue) immunostaining in the hippocampal DG of WT mice and TG mice. Arrows pointed to the neurons. Scale bar, 50 µm. G) Quantitative analysis of RagA and NeuN. Fluorescence intensity of RagA and NeuN in panel F was measured using a densitometric method (n = 5). H) Representative images of TUNEL (red) and DAPI (blue) staining in the hippocampal DG of WT mice and TG mice. Arrows pointed to the TUNEL‐positive apoptotic cells. Scale bar, 50 µm. I) Representative images of TUNEL (red) and DAPI (blue) staining in the hippocampal CA1 of WT mice and TG mice. Arrows pointed to the TUNEL‐positive apoptotic cells. Scale bar, 50 µm. J) Quantitative analysis of TUNEL‐positive apoptotic cells. Fluorescence intensity of TUNEL‐positive apoptotic cells in panel H was measured using a densitometric method (n = 3). K) Quantitative analysis of TUNEL‐positive apoptotic cells. Fluorescence intensity of TUNEL‐positive apoptotic cells in panel I was measured using a densitometric method (n = 3). The data were presented as means ± SD and analysed by unpaired t test. * *p* < 0.05.

Brain specific RagA knock‐in mice were generated and crossed with wild type (WT) C57BL/6N mice to yield heterozygous RagA transgenic mice. The layout of the animal experiments is illustrated in **Figure** **1**A. To localize the overexpression of RagA in the brain regions, the prefrontal cortex (PFC) and hippocampus were recovered from RagA transgenic mice and detected for the protein expression of RagA by Western blotting. Figure 1B,C shows that RagA transgenic mice showed significant overexpression of RagA in the PFC compared with WT mice (t = 3.041, df = 3.412, *P* = 0.0472) and did not show significant changes in the hippocampus (t = 0.7207, df = 3.288, *P* = 0.5190). Consistently, immunofluorescence analysis in Figure 1D,E shows that RagA was significantly upregulated in the PFC from RagA transgenic mice compared with WT mice (t = 3.311, df = 3.996, *P* = 0.0297). As the control, neuronal biomarker NeuN was found to be similar in the PFC from both RagA transgenic mice and WT mice (t = 0.9108, df = 2.456, *P* = 0.4428). Importantly, RagA and NeuN were well co‐localized in the frontal cortex from RagA transgenic mice. By contrast, Figure 1F,G shows that NeuN was significantly reduced in the dentate gyrus (DG) from RagA transgenic mice compared with WT mice (t = 2.903, df = 8, P = 0.0198), whereas RagA was not significantly changed in the DG from RagA transgenic mice and WT mice (t = 0.1214, df = 8, P = 0.9064). Terminal deoxynucleotidyl transferase dUTP nick end labeling (TUNEL) assay in Figure 1H–K showed that the number of TUNEL‐positive apoptotic cells was significantly increased in the DG (t = 4.127, df = 4, P = 0.0145) and cornu ammonis 1 (CA1) (t = 2.842, df = 4, P = 0.0468) from RagA transgenic mice. These results validated that RagA was mainly knocked into the PFC while the decline of NeuN might indicate the loss of neurons in the DG. It is possible that RagA overexpression induced neuronal apoptosis in the DG and CA1.

### 2.2 RagA Induced the Depressive‐Like Behaviors and Memory Impairment

**Figure 2:**
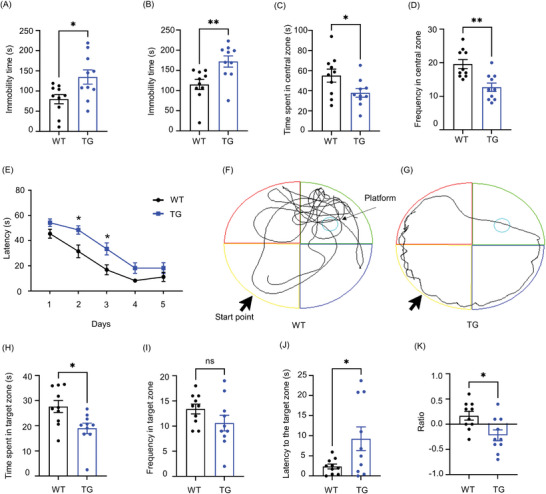
Depressive‐like behaviors and memory impairments were linked to RagA overexpression. A) Immobility time in tail suspension test. B) Immobility time in forced swim test. C) Time spent in central zone of open field test. D) Frequency in central zone of open field test. E) Water maze latency. The data were presented as means ± SEM and analysed by two‐way ANOVA followed by Šídák's multiple comparisons test (n = 10). * *p* < 0.05. F,G) Representative tracks for the behaviors of WT mice and TG mice in the water maze. H) Time spent in target zone of water maze. I) Frequency in target zone of water maze. J) Latency to the target zone of water maze. K) The discrimination index of novel object recognition test. The data were presented as means ± SEM and analysed by unpaired t test (n = 10). * *p* < 0.05; ** *p* < 0.01.

To assess the effects of RagA overexpression on behaviors, first, RagA transgenic mice were assessed for the depressive‐like behaviors by tail suspension test (TST), forced swim test (FST), and open field test (OFT) as previously described.^[^ ^14^ ^]^ TST and FST in **Figure** **2**A,B demonstrated that RagA transgenic mice showed longer immobile time, indicating depressive‐like behaviors, compared with WT mice, TST with t = 2.593, df = 18, *P* = 0.0184; FST with t = 3.063, df = 18, *P* = 0.0067, respectively. OFT in Figure 2C,D showed that RagA transgenic mice spent significantly less time in the central zone of the chamber (t = 2.214, df = 15.61, *P* = 0.0421), and traveled less frequently in the central zone of the chamber than WT mice (t = 3.789, df = 17.90, *P* = 0.0014). Second, the spatial learning and memory of RagA transgenic mice were evaluated by water maze test. Figure 2E demonstrates that RagA transgenic mice significantly extended the latency to the platform in the training trial on Day‐2 (t = 3.162, df = 90, *P* = 0.0106) and Day‐3 (t = 3.078, df = 90, *P* = 0.0137) compared with WT mice. In the probe trial as shown in Figure 2F–H, RagA transgenic mice explored the target zone significantly less than WT mice (t = 2.752, df = 17.70, *P* = 0.0133). In the probe trial as shown in Figure 2I, RagA transgenic mice and WT mice traveled to the target zone in a similar frequency (t = 1.527, df = 15.05, *P* = 0.1476). However, Figure 2J demonstrated that RagA transgenic mice significantly extended the latency to the target zone compared with WT mice (t = 2.298, df = 9.756, *P* = 0.0451). Third, the recognition memory of RagA transgenic mice was assessed by a novel object recognition test. Figure 2K shows that RagA transgenic mice received a negative discrimination index, which was significantly different from that for WT mice (t = 2.807, df = 17.20, *P* = 0.0120). Collectively, these results suggested that RagA transgenic mice exhibited the depressive‐like behaviors and memory impairments compared with WT mice.

### 2.3 RagA Altered Expression of Adenosine A2a Receptor (Adora2a) and Other 48 Genes in Transgenic Mice

**Figure 3:**
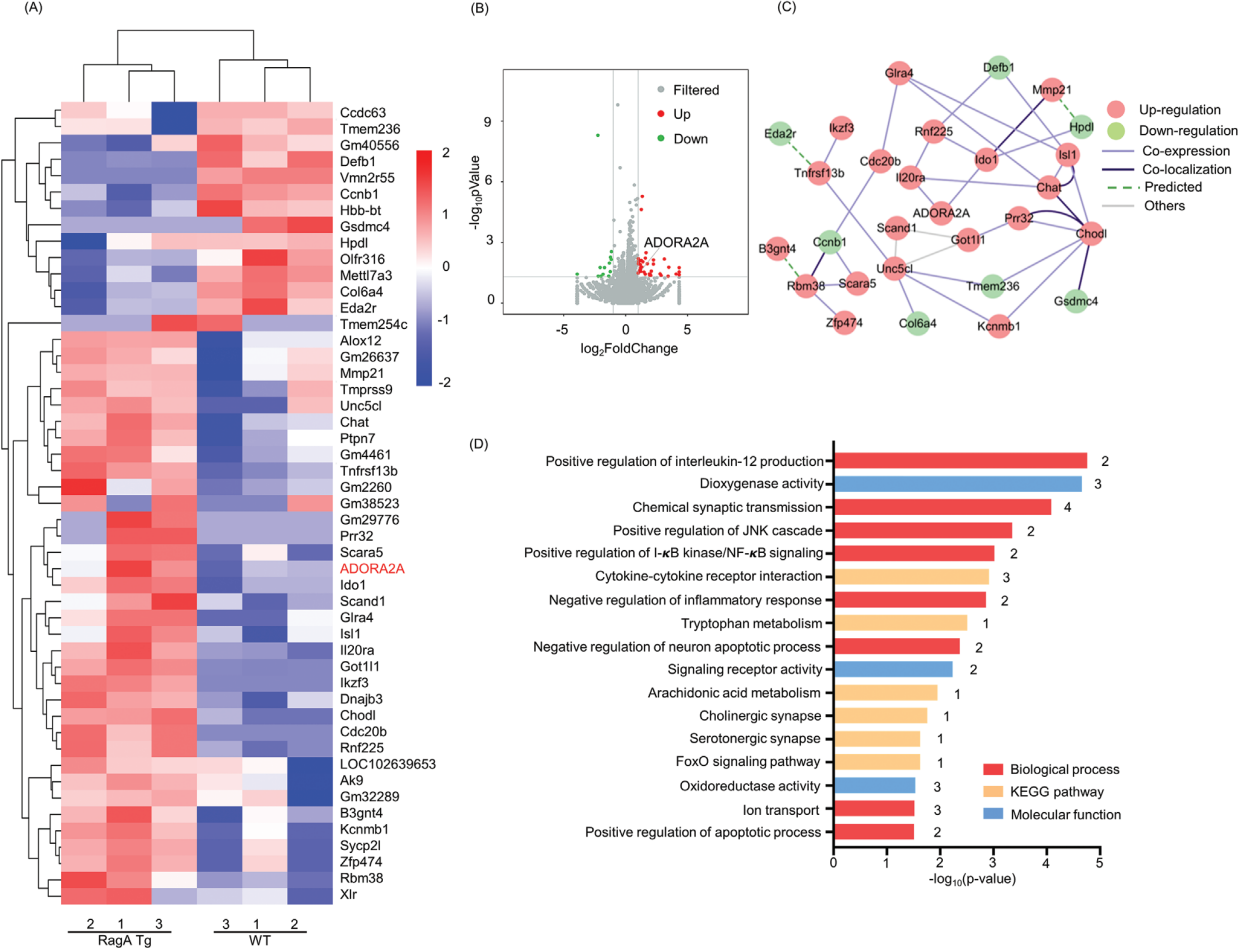
49 differentially expressed genes (DEGs) were identified in the frontal cortex of RagA transgenic mice. A) Heatmap of 49 DEGs. B) Volcano Plot of 36 up‐regulated genes (red), 13 down‐regulated genes (green) and other unchanged genes (grey). C) Interaction network of 21 up‐regulated genes (red) and seven down‐regulated genes (green). D) GO and KEGG pathway enrichment analysis of 49 DEGs.

To investigate the effects of RagA on gene expression in transgenic mice, the total RNAs were isolated from the frontal cortex and profiled by next‐generation RNA sequencing. **Figure** **3**A,B shows that RagA transgenic mice differentially expressed 49 genes (*p* < 0.05 and fold change (FC) ≥ 2), 36 up‐regulated genes, and 13 down‐regulated genes, respectively, compared with WT mice. Figure 3C shows the interaction network involving 21 up‐regulated genes and seven down‐regulated genes. Further Gene Ontology (GO) and Kyoto Encyclopedia of Genes and Genomes (KEGG) pathway enrichment analysis in Figure 3D well annotated the gene functions into eight biological processes (BP), three molecular functions (MF), and six KEGG pathways. Interestingly, these functional groups primarily pertained to inflammation, tryptophan metabolism, and oxidative stress. Interestingly, these results suggested that ADORA2A with fold‐change of 2.15 (*p* = 0.03) might contribute to the dysfunctions in the depressive‐like behaviors.

### 2.4 ADORA2A Was Upregulated in RagA Transgenic Mice

**Figure 4:**
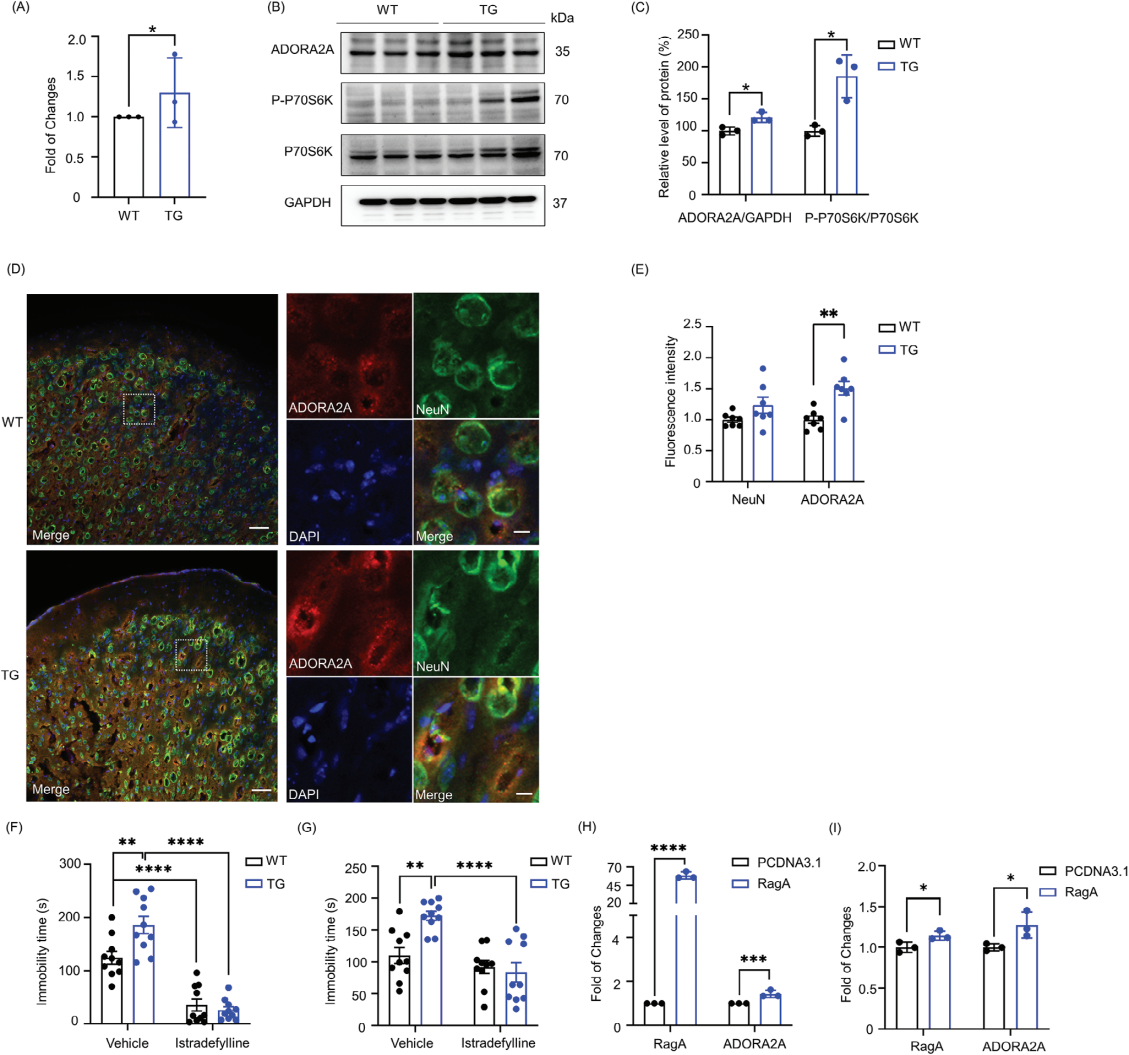
ADORA2A and phospho‐P70S6K (P‐P70S6K) were upregulated in the PFC of RagA transgenic mice. A) ADORA2A mRNA level in the PFC of WT mice and TG mice. B) The representative bands of ADORA2A, P‐P70S6K, and P70S6K in the PFC of WT mice and TG mice. C) Quantitative analysis of ADORA2A, phospho‐P70S6K (P‐P70S6K), and P70S6K. The western blots in panel B were analyzed using a densitometric method (n = 3). D) Representative images of ADORA2A (red), NeuN (green) and DAPI (blue) immunostaining in the PFC of WT mice and TG mice. Scale bar, 50 µm (left) and 10 µm (right). E) Quantitative analysis of ADORA2A and NeuN. Fluorescence intensity of ADORA2A and NeuN in panel D was quantified using a densitometric method (n = 3). The data were presented as means ± SD and analyzed by unpaired t test. * *p* < 0.05. F) Immobility time in tail suspension test. G) Immobility time in the forced swim test. The data were presented as means ± SEM and analyzed by one‐way ANOVA, followed by post hoc Dunnett test (n = 10). * *p* < 0.05; ** *p* < 0.01; *** *p* < 0.001; **** *p* < 0.0001. H) ADORA2A and RagA mRNA levels in the high differentiated PC12 cells (n = 3). The data were presented as means ± SD and analyzed by unpaired t test. *** *p* < 0.001; **** *p* < 0.0001. I) ADORA2A and RagA mRNA levels in the HT‐22 cells (n = 3). The data were presented as means ± SD and analyzed by unpaired t test. * *p* < 0.05.

To verify the association of ADORA2A with RagA overexpression, first, the total RNAs were isolated from the PFC and quantified by quantitative reverse transcriptase polymerase chain reaction (qRT‐PCR). **Figure** **4**A shows that ADORA2A was significantly upregulated in RagA transgenic mice compared with WT mice (t = 2.387, df = 8.000, *P* = 0.0441). Second, Western blot analysis of the PFC in Figure 4B,C demonstrated that the protein level of ADORA2A was significantly elevated in RagA transgenic mice compared with WT mice (t = 3.608, df = 3.704, *P* = 0.0257). On the other hand, P70S6K was also determined as a representative downstream target of RagA. As a result, the levels of phospho‐P70S6K were significantly increased in RagA transgenic mice compared with WT mice (t = 4.268, df = 2.255, *P* = 0.0409). Third, the localization of ADORA2A in the brain was examined by immunofluorescence analysis. Figure 4D,E demonstrates that the ADORA2A specific fluorescence signal was significantly enhanced in the PFC of RagA transgenic mice compared with that of WT mice (t = 4.089, df = 12, P = 0.0015), whereas NeuN specific fluorescence signal was not significantly changed (t = 1.722, df = 12, 0.1106). These results demonstrated that ADORA2A was not only upregulated in the PFC of RagA transgenic mice, but also colocalized with the neuronal biomarker NeuN.

To clarify the role of ADORA2A in depression, RagA transgenic mice were treated with ADORA2A inhibitor Istradefylline and assessed for depressive‐like behaviors. As a result, TST in Figure 4F showed that RagA transgenic mice extended the immobility time and showed a depressive‐like behavior in TST compared with WT mice (F^(3, 36)^ = 40.82, *P* = 0.0024), whereas Istradefylline at the dose of 3 mg k^−1^g significantly decreased the immobility time and attenuated the depressive‐like behaviors in RagA transgenic mice (F ^(3, 36)^ = 40.82, *P* <0.0001) as well as WT mice (F ^(3, 36)^ = 40.82, *P* <0.0001) compared with vehicle. FST in Figure 4G showed that RagA transgenic mice extended the immobility time and showed a depressive‐like behavior in FST compared with WT mice (F ^(3, 36)^ = 11.82, *P* = 0.0017), whereas Istradefylline (3 mg k^−1^g) significantly decreased the immobility time and attenuated the depressive‐like behaviors in RagA transgenic mice (F ^(3, 36)^ = 11.82, *P* <0.0001) compared with vehicle. FST also showed that Istradefylline (3 mg k^−1^g) did not significantly decrease the immobility time in WT mice (F ^(3, 36)^ = 11.82, *P* = 0.5598) compared with vehicle. Collectively, RagA transgenic mice exhibited depressive‐like behaviors via ADORA2A‐dependent mechanism.

To verify the effect of RagA overexpression on ADORA2A expression, highly differentiated PC12 cells and HT‐22 cells were transfected with RagA cDNA expression plasmid and examined for ADORA2A expression. As a result, Figure 4H shows that RagA‐transfected PC12 cells showed significant upregulation of RagA mRNA (t = 38.70, df = 8.000, *P* <0.0001) and ADORA2A mRNA expression (t = 5.072, df = 8.000, *P* = 0.0010) compared with vector‐transfected cells. Figure 4I shows that RagA mRNA (t = 2.925, df = 4, *P* = 0.0430) and ADORA2A mRNA (t = 2.878, df = 4, *P* = 0.0451) levels were also significantly upregulated in HT‐22 cells after RagA transfection. Thus, ADORA2A expression was increased in the RagA‐transfected PC12 cells and HT‐22 cells.

### 2.5 RagA Reduced the Generation of Depression‐Related Metabolites and Neurotransmitters

**Figure 5:**
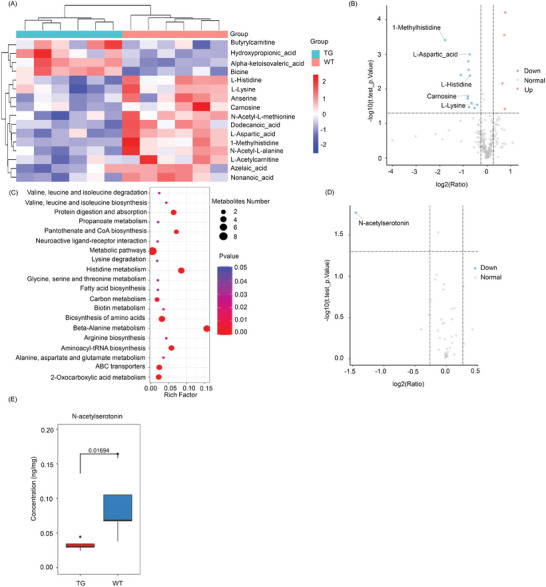
16 differentially regulated metabolites (DRMs) were identified in the frontal cortex of RagA transgenic mice. A) Heatmap of 16 DRMs. B) Volcano Plot of four up‐regulated metabolites (red), 12 down‐regulated metabolites (blue), and other unchanged metabolites (grey). C) KEGG pathway enrichment analysis of 16 DRMs. D) Volcano Plot of one down‐regulated neurotransmitter (blue) and other unchanged neurotransmitters (grey). E) Concentration of N‐acetylserotonin in the frontal cortex of WT mice and TG mice. The data were presented as median, first quantile, third quantile and analyzed by unpaired t test. *P* = 0.01694.

To examine the effects of RagA on metabolite profiles, the PFC tissues were analyzed by ultra‐performance liquid chromatography tandem mass spectrometry (UPLC‐MS/MS)‐based metabolomics techniques. As a result, **Figure** **5**A,B shows that 16 metabolites were differentially detected in RagA transgenic mice and WT mice at the ratio of ≥ 1.2 or ≤ 0.83 (*p* < 0.05), specifically, four up‐regulated and 12 down‐regulated. Online biological annotation suggested that five down‐regulated metabolites (i.e., L‐methylhistidine, L‐aspartic_acid, L‐histidine, carnosine and L‐lysine) could be associated with depression. L‐Methylhistidine was mostly downregulated in RagA transgenic mice (ratio = 0.29, *p* = 3.85E‐04). The metabolic pathway enrichment analysis of differentially regulated metabolites (DRMs) was performed. KEGG pathway enrichment analysis showed that selected DRMs could be enriched into 20 KEGG pathways in Figure 5C. The metabolites were primarily enriched to beta‐alanine metabolism (*p* = 1.43E‐11), histidine metabolism (*p* = 2.58E‐08), and biosynthesis of amino acids (*p* = 1.49E‐06). These results suggested that RagA overexpression might disrupt the metabolic homeostasis to trigger depressive‐like behaviors.

To examine the effects of RagA on different neurotransmitters, the PFC tissues were analyzed by UPLC‐MS/MS‐based metabolomics techniques. A total of 39 neurotransmitters were screened against the criteria: ratio, ≥ 1.2 or ≤ 0.83; *p*, < 0.05. Figure 5D,E shows that N‐acetylserotonin was downregulated in the PFC of RagA transgenic mice compared with that of WT mice (ratio = 0.37, *p* = 0.01694). These results suggested that RagA overexpression highly affected depression‐related neurotransmitters.

### 2.6 RagA Overexpression Altered the Expression of 13 Depression‐Related Proteins

**Figure 6:**
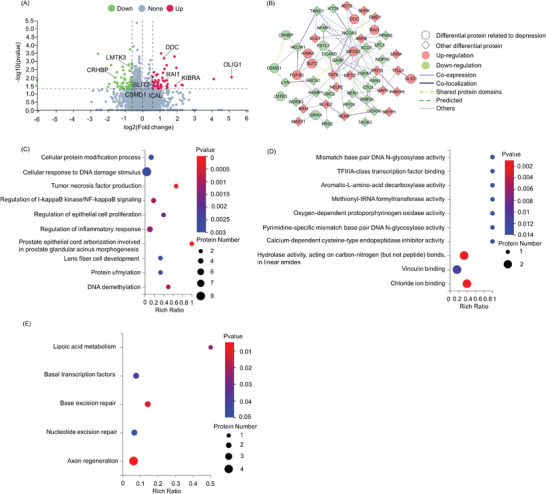
108 differentially expressed proteins (DEPs) were identified in the PFC of RagA transgenic mice. A) Volcano Plot of 46 up‐regulated proteins (red), 62 down‐regulated proteins (green) and other unchanged proteins (grey). B) Interaction network of up‐regulated genes (red) and down‐regulated genes (green). C) GO enrichment analysis of 108 DEPs in terms of “BP”. D) GO enrichment analysis of 108 DEPs in terms of “MF”. E) KEGG pathway enrichment analysis of 108 DEPs.

To investigate the effects of RagA on the proteome profile, the PFC tissues were collected from RagA transgenic mice and WT mice and analyzed by data‐independent acquisition (DIA) quantification proteomics. **Figure** **6**A showed that 108 differentially expressed proteins (DEPs) (*p* < 0.05 and FC>1.5) were identified, 46 up‐regulated proteins and 62 down‐regulated proteins, respectively. Online database search revealed that 13 DEPs (i.e., OLIG1, KIBRA, RAI1, FGFR2, DDC, MED22, SLIT2, LMTK3, CSMD1, COX3, FSTL1, GRIK4, CRHBP) were associated with depression. OLIG1 was upregulated to the largest extent (FC = 35.53, *p* = 0.009, RagA transgenic mice vs WT mice). Figure 6B shows the interaction network involving seven up‐regulated proteins and six down‐regulated proteins. The GO and KEGG pathway enrichment analysis in Figure 6C–E showed that the selected DEPs were enriched into 10 BP, 10 MF, and 5 KEGG pathways. These selected proteins could be predominantly annotated into the functional groups of protein modification, cellular response to DNA damage and inflammation.

### 2.7 DEPs and DRMs Were Correlated with the Pathology of Depression

**Figure 7:**
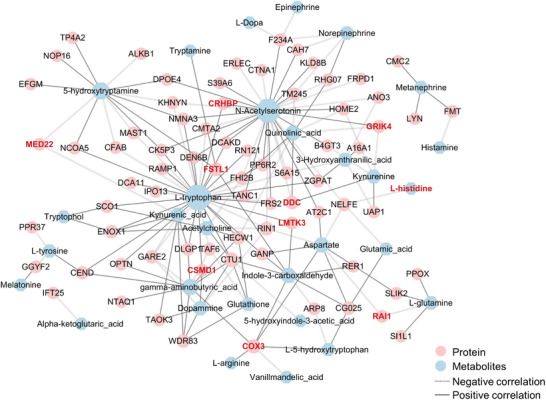
Proteomics was correlated with metabolomics in RagA transgenic mice.

To investigate the interactions between DEPs and DRMs, the selected proteins and metabolites were mapped for different functional interaction networks and analyzed for Spearman correlation. As a result, **Figure** **7** shows that the key metabolomic variables included N‐acetylserotonin, L‐tryptophan, and 5‐hydroxytryptamine whereas the key proteomic variables included GRIK4, DDC, CRHBP, MED22, ENOX1, CSMD1, and LMTK3. Specifically, N‐acetylserotonin had a positive correlation with GRIK4 and CRHBP, and a negative correlation with DDC; L‐Tryptophan was positively correlated with ENOX1, CSMD1 and LMTK3; 5‐Hydroxytryptamine was negatively correlated with MED22 (see details in Table S1, Supporting information). These results suggested that RagA overexpression coordinately altered the depression‐regulating proteins and metabolites.

Interaction network of DEPs and DRMs. The proteins and neurotransmitters (red font) were related to depression.

### 2.8 A Total of 195 Differentially Expressed Long Non‐Coding RNAs (lncRNAs) Including TCONS_00004755 Were Found in RagA Transgenic Mice

**Figure 8:**
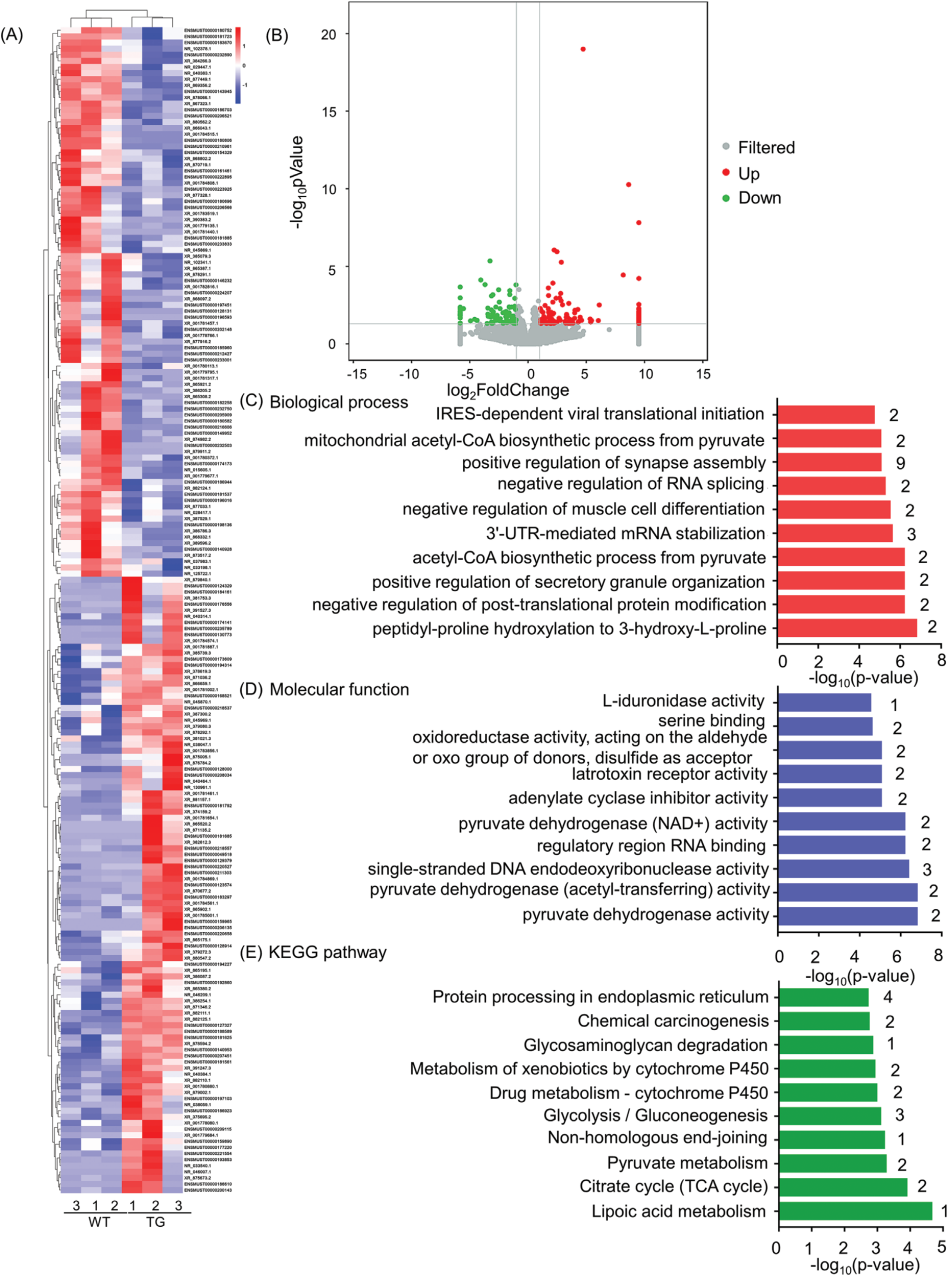
Detection of differentially expressed lncRNAs in the prefrontal cortex of RagA transgenic (TG) mice and WT mice. A) Heatmap of 195 differentially expressed lncRNAs. B) Volcano Plot of 104 up‐regulated lncRNAs (red), 91 down‐regulated lncRNAs (green) and other unchanged lncRNAs (grey). C) GO enrichment analysis of 195 differentially expressed lncRNAs in terms of “BP”. D) GO enrichment analysis of 195 differentially expressed lncRNAs in terms of “MF”. E) KEGG pathway enrichment analysis of 195 differentially expressed lncRNAs.

To investigate the effects of RagA on lncRNAs expression in transgenic mice, the total RNAs were isolated from the frontal cortex and profiled by next‐generation RNA sequencing technology. **Figure** **8**A,B shows that RagA transgenic mice differentially expressed 195 lncRNAs (*p* < 0.05 and fold change ≥ 2), 104 up‐regulated lncRNAs, and 91 down‐regulated lncRNAs, respectively. TCONS_00004755 (FC = 730.50, *p* = 0.00006) and XR_865308.2 (FC = 0.02, *p* = 0.0056) were the most up–regulated and down–regulated lncRNA, respectively. GO and KEGG pathway enrichment analysis in Figure 8C–E well annotated the functions of these lncRNAs into ten BP, ten MF, and ten KEGG pathways. These results suggest that the lncRNAs in the positive regulation of synapse assembly were the most affected.

## 3 Discussion

The increasing prevalence and negative social impact of depression are strong signals for the need of effective treatments while the side effects of the existing antidepressant drugs constitute the challenge for the discovery of new targets and therapies.^[^ ^1b^ ^]^ The bidirectional interaction between inflammation and depression indicates the need for new dual anti‐inflammatory and antidepressant drugs.^[^ ^3b^ ^]^ Indeed, bacterial LPS induced neuroinflammatory responses and depressive‐like behaviors in mice.^[^ ^15^ ^]^ Importantly, LPS also upregulated RagA expression in mice and neurons, suggesting a role of RagA in depression.^[^ ^6^, ^7^ ^]^ Small GTPase RagA not only senses the availability of intracellular amino acids for mTORC1 signaling, but also physically interacts with different mTORC1 components.^[^ ^16^ ^]^ Although the regular knock‐in of RagA is lethal, in the present study, RagA was successfully overexpressed in the brains of mice. RagA transgenic mice were characterized by multiple omics technologies to gain molecular insights into the potential of RagA as a novel therapeutic target against depression.

Dysregulation of mTORC1 signaling is associated with human neurological disorders, including mood disorders, mental retardation syndromes, autism spectrum disorders, and memory impairment.^[^ ^7^, ^8^, ^17^ ^]^ Hippocampal volume was significantly reduced in the patients with major depressive disorder compared with people without depression.^[^ ^18^ ^]^ In this study, we found that brain‐specific RagA overexpression caused depressive‐like behaviors and memory deficit (Figure 2A–K). Presumably, RagA affects the relevant neuropsychiatric processes primarily through mTORC1‐mediated mechanisms.^[^ ^6^ ^]^ However, RagA was mainly overexpressed in the PFC and not significantly increased in the hippocampus of RagA transgenic mice (Figure 1B–E). DG is known to exhibit a multitude of plasticity mechanisms for regulating learning and memory functions and encompassing the continuous generation of new neurons.^[^ ^19^ ^]^ It is difficult to explain why neurons were lost in the DG of RagA transgenic mice (Figure 1F,G). TUNEL assay suggested that RagA knock‐in induced apoptosis in the DG and CA1 (Figure 1H–K), ultimately leading to the neuronal loss. By searching for DEGs in the PFC of RagA transgenic mice, we identified 49 DEGs including ADORA2A in RagA transgenic mice compared with WT mice (Figure 3A–C). These genes were mainly enriched to the functional groups of inflammation, tryptophan metabolism, serotonergic synapse, and oxidative stress (Figure 3D). ADORA2A was increased by the fold‐change of 2.15 (*p* = 0.03). ADORA2A is a G protein‐coupled receptor and binds adenosine with a high affinity.^[^ ^20^ ^]^ ADORA2A mainly exists on the presynaptic terminals in the brain and modulates the development of dendrite branching and the process of axonal elongation.^[^ ^21^ ^]^ ADORA2A is upregulated in conditions such as depression, neuroinflammatory diseases, and neurodegenerative diseases.^[^ ^22^ ^]^ The overactivation of ADORA2A leads to the release of glutamate through regulation of the protein kinase A (PKA)/cyclic adenosine monophosphate (cAMP)/cAMP response element binding protein (CREB) signaling pathway, resulting in cognitive disorders.^[^ ^22^, ^23^ ^]^ ADORA2A also regulates synaptic activity and is increased in the mesolimbic pathways.^[^ ^12^ ^]^ Indeed, ADORA2A is a new target for the development of antidepressant drugs.^[^ ^24^ ^]^ ADORA2A antagonists could alleviate depressive symptoms and anxiety‐related behaviors.^[^ ^25^ ^]^ For instance, the ADORA2A antagonist KW6002 attenuated chronic restraint stress‐induced depressive‐like behaviors in mice.^[^ ^26^ ^]^ ADORA2A inhibitor Istradefylline exhibited anti‐depressant activities in a rat learned helplessness model.^[^ ^27^ ^]^ In this study, we demonstrated that ADORA2A and phospho‐P70S6K were upregulated in the PFC of RagA transgenic mice (Figure 4A–C). ADORA2A was detected in the neurons in the PFC of RagA transgenic mice (Figure 4D,E). Others also found that ADORA2A was expressed in the striatopallidal neurons.^[^ ^28^ ^]^ In this study, we not only found the upregulation of ADORA2A and phospho‐P70S6K, but also detected ADORA2A in the neuron in the PFC of RagA transgenic mice (Figure 4). Interestingly, previous studies also found ADORA2A in the striatopallidal and hippocampal neurons.^[^ ^21^, ^28^ ^]^ Others discovered that ADORA2A was detected in the neurons other than the astrocytes, oligodendrocytes, and microglia from aging human brains and Alzheimer's disease brains.^[^ ^22c^ ^]^ Importantly, ADORA2A antagonist Istradefylline significantly decreased the immobility time and attenuated the depressive‐like behaviors in the RagA transgenic mice (Figure 4F,G). Moreover, RagA transfection significantly upregulated ADORA2A mRNA in PC12 cells and HT‐22 cells compared with vector transfection (Figure 4H,I). Taken together, these results consolidated that the dynamic link between RagA and ADORA2A might play a role in depression although little is known about the mechanism by which RagA overexpression induces ADORA2A.

Genetic and environmental factors are known to alter the profiles of metabolites related to depression.^[^ ^29^ ^]^ In the present study, we applied metabolomics to identify the DRMs and neurotransmitters in the PFC of RagA transgenic mice. Among 16 DRMs in Figure 5A, five depression‐associated DRMs including L‐methylhistidine, L‐aspartic_acid, L‐histidine, carnosine, and L‐lysine were down‐regulated (Figure 5B). These regulated metabolites were enriched into the primary pathways of beta‐alanine metabolism, histidine metabolism, and biosynthesis of amino acids (Figure 5C). L‐Histidine is required for multiple biological functions such as protein synthesis, coordination of metal ions, and elimination of reactive oxygen and nitrogen species.^[^ ^30^ ^]^ L‐Histidine reduction is associated with moderate depressive symptoms.^[^ ^31^ ^]^ As a derivative of L‐histidine by N1‐methylation, L‐methylhistidine was similarly reduced in the post‐stroke depression patients compared with healthy subjects.^[^ ^32^ ^]^ L‐Aspartic acid is biosynthesized in the neuroendocrine tissues of humans and acts as an excitatory neurotransmitter in the central nervous system.^[^ ^33^ ^]^ The lower levels of L‐aspartic acid were detected in the brains of depressed animal models.^[^ ^34^ ^]^ Carnosine is an essential endogenous dipeptide and exhibits anti‐inflammatory and immunomodulatory properties.^[^ ^35^ ^]^ The serum levels of carnosine were lower in the major depressive disorder patients compared to the non‐depressed controls.^[^ ^36^ ^]^ L‐Lysine not only contributes to the production of hormones, antibodies, and enzymes, but also serves as a substrate for the biosynthesis of carnitine for regulating lipid metabolism.^[^ ^37^ ^]^ The levels of L‐lysine were downregulated in the animal models of depression.^[^ ^34^ ^]^ With respect to differentially regulated neurotransmitters (DRNs), we found that N‐acetylserotonin was downregulated in the PFC of RagA transgenic mice compared with WT mice (Figure 5D,E). It is well‐known that monoaminergic neurotransmitters (i.e., serotonin, norepinephrine, dopamine) are commonly dysregulated in depression.^[^ ^38^ ^]^ In the biosynthesis of neurotransmitters, L‐ tryptophan is hydroxylated to form 5‐hydroxytryptophan (5‐HTP) by L‐tryptophan‐5‐hydroxylase while 5‐HTP is decarboxylated to form serotonin.^[^ ^39^ ^]^ Serotonin is further acetylated to form N‐acetylserotonin by arylalkylamine N‐acetyltransferase.^[^ ^40^ ^]^ N‐Acetylserotonin is a precursor of melatonin and modulates the circadian rhythms.^[^ ^41^ ^]^ N‐Acetylserotonin was shown to improve depressive behaviors by regulating tyrosine kinase (TrkB) receptors.^[^ ^42^ ^]^ Indeed, the current study found that N‐acetylserotonin and several DEGs were enriched into tryptophan metabolism. As for the reduction of DRMs and DRNs, indoleamine 2,3‐dioxygenase 1 (IDO1) is the key upregulated DEG in RagA transgenic mice. IDO1 catalyzes L‐tryptophan catabolism to kynurenine and consequently limits the availability of L‐tryptophan for the generation of neurotransmitters like serotonin and melatonin.^[^ ^43^ ^]^ N‐acetylserotonin directly binds to IDO1 and enhances IDO1 activity in vitro and in vivo.^[^ ^44^ ^]^ Ultimately, RagA overexpression reduced several key depression‐related neurotransmitters and metabolites in transgenic mice.

Proteomic profiles of brain tissues greatly facilitate the elucidation of the mechanisms underlying the pathology of various diseases.^[^ ^45^ ^]^ To understand the pathologic impact of RagA overexpression, we also applied UPLC‐MS/MS‐based proteomics technology to identify the DRPs in the PFC of RagA transgenic mice. As a result, we identified 108 DRPs from RagA transgenic mice (Figure 6A). Of thirteen depression‐associated DRPs, seven proteins including DDC, FGFR2, KIBRA, MED22, OLIG1, RAI1, SLIT2 were upregulated, whereas six proteins including COX3, CRHBP, CSMD1, FSTL1, GRIK4, and LMTK3 were downregulated (Figure 6B). These DRPs were enriched into the primary functional groups of protein modification, response to DNA damage, inflammation, and transcription (Figure 6C–E). COX3 representing cytochrome c oxidase subunit III encodes the catalytic subunits of the cytochrome c oxidase enzyme.^[^ ^46^ ^]^ It was reported that LPS administration might induce depressive‐like behaviors in rats by downregulating COX3 mRNA.^[^ ^47^ ^]^ CRHBP representing glycoprotein corticotropin‐releasing hormone (CRH) binding protein regulates the interaction between CRH and CRH receptors.^[^ ^48^ ^]^ The mRNA levels of CRHBP were significantly downregulated in the patients with major depressive disorder.^[^ ^49^ ^]^ CSMD1 representing CUB and Sushi multiple domains 1 modulates the complement activation and inflammatory responses in the central nervous system.^[^ ^50^ ^]^ CSMD1 KO mice exhibit depressive‐like behaviors.^[^ ^51^ ^]^ OLIG1 representing oligodendrocyte transcription factor 1 is persistently expressed during the development of the oligodendrocyte lineage cells but elevated in the patients with major depressive disorder.^[^ ^52^ ^]^ RAI1 representing retinoic acid‐induced 1 is a nucleosome‐binding protein, expressed in the brain, and involved in the regulation of neuronal communication.^[^ ^53^ ^]^ RAI1 is elevated in the brains of patients with major depression.^[^ ^54^ ^]^ SLIT2 representing slit guidance ligand 2 instructs axon growth and neuronal progenitor cell migration in the nervous system.^[^ ^55^ ^]^ SLIT2 overexpression induces depression‐ and anxiety‐like behaviors in mice.^[^ ^56^ ^]^ In terms of disease‐gene association, the upregulation of OLIG1, RAI1, SLIT2 and the downregulation of COX3, CRHBP, CSMD1 in the RagA transgenic mice were positively correlated with depression. By contrast, the upregulation of DDC, FGFR2, KIBRA, and MED22, and the downregulation of FSTL1, GRIK4, and LMTK3 in the RagA transgenic mice were negatively correlated with depression. DDC representing DOPA decarboxylase is a rate‐limiting enzyme in dopamine synthesis.^[^ ^57^ ^]^ The activity and concentration of DCC were reduced in the serum of patients with non‐psychotic major depression and psychotic major depression.^[^ ^58^ ^]^ FGFR2 representing fibroblast growth factor (FGF) receptor 2 is a membrane‐spanning tyrosine kinase that regulates FGF signaling in neurite outgrowth and hippocampal neurogenesis.^[^ ^59^ ^]^ FGFR2 is decreased in the brain of major depressive disorder patients.^[^ ^60^ ^]^ KIBRA representing kidney and brain‐expressed protein is a scaffolding protein, which exists at the postsynaptic regions in the neural synapses, and regulates synaptic plasticity and memory.^[^ ^61^ ^]^ The serum level of KIBRA is decreased in the patients with depression.^[^ ^62^ ^]^ MED22 representing mediator complex subunit 22 is a subunit of the mediator complex that acts as a coactivator for gene transcription in the eukaryotes.^[^ ^63^ ^]^ MED22 was downregulated in the major depressive disorder patients with emotional neglect in childhood.^[^ ^64^ ^]^ FSTL1 representing follistatin‐like protein 1 is a secreted glycoprotein and regulates the inflammation and metabolism.^[^ ^65^ ^]^ FSTL1 was increased in the hippocampus of chronic unpredictable mild stress exposed mice.^[^ ^66^ ^]^ GRIK4 representing glutamate receptor ionotropic kainate 4 encodes a high affinity kainate receptor subunit in the brain.^[^ ^67^ ^]^ The mice overexpressing GRIK4 in the forebrain exhibited the depressive behaviors.^[^ ^68^ ^]^ LMTK3 representing lemur tyrosine kinase 3 acts as a client protein for heat shock protein 90.^[^ ^69^ ^]^ Lmtk3^−/−^ mice exhibited less depression‐like behaviors compared with WT mice.^[^ ^70^ ^]^ Such discrepancy may be resulted from the accuracy of LC‐MS/MS detection and the overall impact of different DRPs on the pathology of depression.

RagA overexpression likely alters the interaction dynamics of gene expression, protein profiles, and the biosynthesis of metabolites and neurotransmitters to induce depressive‐like behaviors and memory impairment in transgenic mice. We performed the correlation analysis to understand the relationships between different omics results as previously described.^[^ ^71^ ^]^ The interaction network and Pearson correlation coefficient in Figure 7 and Table S1 (Supporting Information) showed: 1) N‐acetylserotonin, GRIK4 and CRHBP were positively correlated; 2) L‐Tryptophan, ENOX1, CSMD1 and LMTK3 were positively correlated; 3) N‐acetylserotonin and DDC were negatively correlated; 4) 5‐Hydroxytryptamine and MED22 were negatively correlated. As an example, DDC not only catalyzes the decarboxylation of aromatic amino acid including DOPA but also transforms 5‐HTP to serotonin.^[^ ^72^ ^]^ Serotonin is converted to N‐acetylserotonin by serotonin N‐acetyltransferase for regulating mode and circadian clock.^[^ ^73^ ^]^ Furthermore, 195 differentially expressed lncRNAs including TCONS_00004755 were identified in RagA transgenic mice while WT mice were used as control (Figure 8A,B). These lncRNAs were predominantly enriched to the positive regulation of synapse assembly based on functional annotation (Figure 8C–E). Although lncRNAs are not translated into proteins, these RNA molecules with the length of more than 200 nucleotides are known to influence different BP.^[^ ^74^ ^]^ LncRNAs regulate neuroinflammation and synaptic plasticity in the central nervous system.^[^ ^75^ ^]^ LncRNAs were abnormally expressed in the brain and blood of patients with depression.^[^ ^76^ ^]^ Moreover, LncRNAs serve as the biomarkers for depression severity and therapeutic responsiveness.^[^ ^77^ ^]^ Collectively, these results confirmed that RagA overexpression disrupted the transcriptomic, proteomic, and metabolomic associations with depression in mice.

## 4 Conclusion

The present study established and validated a brain specific RagA knock‐in mouse model of depression. RagA transgenic mice exhibited depressive‐like behaviors and suffered from hippocampal neuron loss and memory impairment. RagA overexpression in the PFC not only altered the expression of genes, lncRNAs and proteins in the PFC region, but also affected the generation of metabolites and neurotransmitters in the whole brain. The DRPs and DRMs/DRNs revealed the transcriptomic, proteomic, and metabolomic associations between RagA overexpression and depression phenotype. Importantly, the activation of the p70S6K/ADORA2A signaling pathway might be a key mechanism underlying the depression of RagA transgenic mice. Therefore, the current study identified the RagA‐ADORA2A pathway as an important target for the development of new antidepressant drugs.

## 5 Experimental Section

### Animals and Drug Administration

Animal experiments were performed following the protocols in CULATR 4762‐18, which was approved by the University's Committee on the Use of Live Animals in Teaching and Research. Adult WT C57BL/6N mice, aged 8–12 weeks, were obtained from the Centre for Comparative Medicine Research (CCMR) at the University of Hong Kong (HKU). Brain specific RagA knock‐in mice were initially generated by Cyagen Biosciences (8‐12 weeks of age, Guangzhou, China) and maintained by the CCMR at HKU. Regular knock‐in of RagA appeared to cause early death in mice.^[^ ^13^ ^]^ To prevent the perinatal lethality, RagA knock‐in mice were bred with WT C57BL/6N mice to generate heterozygous RagA transgenic mice for the current study. SYN1 promoter was used to overexpress the mRraga gene in mice. The piggybac transposon gene expression vector was used to construct the transgene. RagA transgenic mice were generated by crossing RagA knock‐in mice with WT C57BL/6N mice. Male RagA transgenic mice were used in these experiments for depressive‐like behaviors. Mice were accommodated in an environment with regulated humidity and temperature, following a 12‐h light‐dark cycle, and were provided unrestricted access to standard laboratory mice food and water. During the behavioral tests, each mouse was tested individually to avoid any potential carry‐over effects. For drug administration, Istradefylline (3 mg k^−1^g) was dissolved in saline with 15% dimethyl sulfoxide and 15% ethoxylated castor oil. Mice were randomly divided into four groups: WT mice+vehicle, RagA transgenic mice+vehicle, WT mice+Istradefylline (3 mg k^−1^g), RagA transgenic mice+ Istradefylline (3 mg k^−1^g). Istradefylline was intragastrically administered to mice while the equal volume of saline with 15% dimethyl sulfoxide and 15% ethoxylated castor oil was administered to control animals. The behaviors of animals were assessed after 1 h Istradefylline treatment.

### Genotyping

Genomic DNA was extracted with TaKaRa MiniBEST Universal Genomic DNA Extraction Kit (TaKaRa, Japan). PCR amplification was conducted at an annealing temperature of 60 °C with 2×Taq Master Mix (Vazyme, Nanjing, China). The primers with the sequences of 5ˊ‐ATGTGCACCGCTTTGAGAA‐3ˊ for RagA‐F and 5ˊ‐GTATTTGTGAGCCAGGGCATTG −3ˊfor RagA‐R were synthesized (Integrated DNA Technologies, Singapore).

### Tail Suspension Test (TST)

Depressive‐like behaviors were evaluated as previously described.^[^ ^14a^ ^]^ Mice were suspended by attaching their tails to a suspension bar using adhesive tape. The distance between the mouse's nose and the floor of the apparatus was set to be 20–25 cm. The tape was applied to the very tip of the tail, leaving 2–3 mm of the tail exposed. The immobility time of the mice within a duration of 6 min was recorded by a blinded observer. Mice were considered immobile if they hung passively without any movement.

### Forced Swim Test (FST)

Depressive‐like behaviors were evaluated as previously described.^[^ ^78^ ^]^ Mice were placed in a transparent acrylic water tank (30 cm in height, 20 cm in diameter), which was filled with clean water to a depth of 15 cm and maintained at a temperature of 23 ± 2 °C. Following a 2‐min acclimation period, a blinded observer recorded the duration of immobility, which was defined as passive floating or absence of movement, during a 4‐min interval.

### Open Field Test (OFT)

OFT was conducted as previously described.^[^ ^79^ ^]^ Each mouse was positioned at the mid‐point of a polymeric cubic chamber (dimensions: 40 cm × 40 cm × 40 cm) for a duration of 6 min. A 13 cm × 13 cm region was designated as the central area. The mouse behavior was recorded via video camera system and subsequently analyzed.

### Water Maze

The spatial memory was assessed as previously described.^[^ ^80^ ^]^ In brief, an open circular pool (diameter: 90 cm, height: 45 cm) was filled with water at 22 ± 2 °C. The pool was divided into four quadrants, while a hidden platform (diameter: 6 cm, height: 39 cm) was placed in one of the quadrants and submerged 1 cm beneath water. The test involved two phases: acquisition trials for 5 days and probe trial for 1 day. During the acquisition trial, mice were trained every day by randomly arranging starting points in the water. The latency to the platform was recorded and averaged daily for data analysis. On day 6, the probe trial was conducted without a platform in the pool. Each mouse was placed into the pool and given 1 min to search the platform. The trajectory of mice in the pool was recorded using SMART system (PanLab, USA). To assess spatial memory, the latency to the target zone, the duration time spent in the target zone, and the crossing number of the target zone were obtained.

### Novel Object Recognition Test (NORT)

NORT was carried out as previously described.^[^ ^81^ ^]^ Each mouse was placed in a chamber (40 cm × 40 cm × 40 cm) for one training session and one testing session. During the training process, each mouse was allowed to explore the chamber with two identical water‐containing plastic cylinders in the opposite corners for 10 min. On the day after the training session, a test session was conducted while a new plastic cylinder with water was placed within the chamber. Exploration was characterized as the exploration of an object at a proximity of less than 2 cm or through direct nasal contact. The discrimination index of the exploration of an object was calculated.

### Next‐Generation RNA‐Sequencing

The PFC (3 mm) was excised from three mouse brains and immediately frozen in liquid nitrogen. The total RNAs were extracted with the TRIzol (Thermo Fisher Scientific, USA). RNA integrity was analyzed using an Agilent 2100, with the RIN values of >7.6. After the removal of rRNAs with RNase H kit (BGI, China), the cDNAs were synthesized from fragmented RNAs, and sequenced on the DNBSEQ System (BGI, China). The clean reads were mapped to the mouse genome (GRCm38) using HISAT2.^[^ ^82^ ^]^ For mRNAs, Cufflinks were used to calculate fragments per kilobase of transcript per million fragments mapped (FPKM) of each gene, while these read counts of each gene were acquired with HTSeq‐count.^[^ ^83^ ^]^ For lncRNAs, the transcriptome from each dataset was assembled independently using the Cufflinks 2.0 program.^[^ ^83a^ ^]^ All transcriptomes were pooled and merged to generate a final transcriptome using Cuffmerge (Cufflinks 2.0). The transcripts longer than 200 bp and the number of exons > 2 were picked out, and the CPC (v 0.9‐r2),^[^ ^84^ ^]^ PLEK (v 1.2),^[^ ^85^ ^]^ CNCI (v 1.0),^[^ ^86^ ^]^ Pfam (v 30)^[^ ^87^ ^]^ were used to predict transcripts with coding potential. The characteristics (including length, type, number of exons) of lncRNA were analyzed after screening. The novel predicted lncRNAs and known lncRNAs (from NCBI and Ensemble database) were both used for expression calculation and differential screening. Differential gene and lncRNA expression analysis was performed with DESeq (2012) R package at Oebiotech, China. A nominal significance threshold of *p* < 0.05 and fold change (FC) ≥ 2 were established for significant differential expression. The heatmap of DEGs and differentially expressed lncRNAs were generated using R package, while the interaction network of these genes was created with Cytoscape. Finally, Gene Ontology (GO) enrichment analysis and the Kyoto Encyclopedia of Genes and Genomes (KEGG) pathway were conducted using R package while *p* < 0.05 was considered significantly enriched for the DEGs and differentially expressed lncRNAs.^[^ ^88^ ^]^

### Metabolomics

Identification of DRMs6975841: The total of 350 metabolites were detected and quantified by Ultra‐performance liquid chromatography tandem mass spectrometry (UPLC‐MS/MS) as previously described.^[^ ^89^ ^]^ Briefly, the PFC (3 mm) was collected from six mouse brains and processed at BGI, China. The mixtures of 20 µL mouse brain sample, 20 µL standard, and 120 µL sample release agents were shaken at 1200 rpm for 20 min. The supernatant (30 µL), derivatization reagent (20 µL) and N‐Ethyl‐N’‐(3‐dimethylaminopropyl)carbodiimide (EDC) working solution (20 µL) were added to a 96‐well plate and then shaken at 1200 rpm for 60 min at 30 °C. Supernatant (100 µL) was mixed with inner standard II working solution (10 µL) in a new 96‐well plate at 650 rpm for 5 min and further analyzed using Waters UPLC I‐Class Plus system (Waters, USA) with a QTRAP 6500 Plus mass spectrometer (SCIEX, USA). The chromatography was performed on a BEH C18 column (2.1 mm x 10 cm, 1.7 µm, Waters, USA) at 40 °C. Solvent A consisted of 0.1% formic acid in water whereas Solvent B consisted of the mixture of acetonitrile and isopropanol (v/v, 7/3). Elution was performed as follows: 0 min, 5% B; 1 min, 5% B; 5 min, 5% B; 9 min, 70% B; 11 min, 50% B; 13.5 min, 22% B; 14 min, 95% B at the flow rate of 0.400 mL/min; 16 min, 100% B at the flow rate of 0.600 ml/min; 18 min, 5% B at the flow rate of 0.400 mL/min. The MS detection employed a QTRAP 6500 Plus with ESI Turbo ion spray interface and used parameters as follows: ion spray voltage: 4500 V (positive mode) and −4500 V (negative mode); ion source temperature: 400  °C; ion source gas 1 (GS1) at 60 psi, ion source gas 2 (GS2) at 60 psi and curtain gas at 35 psi, respectively. The metabolites were identified by the multi‐reaction monitoring (MRM) method with optimized MRM parent‐daughter transition information of target metabolites, collision energy (CE), declustering potential (DP) and retention time. A data matrix containing information such as metabolite identity and quantitative results were obtained with skyline (https://skyline.ms/) while the results in table format were further processed for information analysis. R package metaX was applied for the identification of the DRMs, while the DRMs between two groups were compared by the univariate analysis methods including ratio analysis and t‐test. Metabolites with ratio ≥ 1.2 or ratio ≤ 0.83 and *p* value < 0.05 were considered as DRMs.

### Identification of Differentially Regulated Neurotransmitters

The total of 39 neurotransmitters were identified and quantified by LC‐MS/MS. The samples (50 mg) were homogenized with grinding beads in 400 µL of 50% methanol and centrifuged at 25000 rpm for 2 min. The standard solution was prepared by including 39 kinds of neurotransmitter standards. The mixture of 20 µL sample and 20 µL standard in 60 µL pre‐cooled 50% methanol was shaken at 1200 rpm for 5 min, precipitated for 4 h at ‐20 °C, and centrifuged at 20 000 g for 15 min at 4 °C. The supernatants were analyzed using Waters Iclass‐AB Sciex 6500 HPLC‐MS/MS system. The chromatography was performed using a UPLC BEH C18 column (Waters, 2.1 mm x 10 cm, 1.7 µm) at 45 °C. Solvent A was 0.1% formic acid aqueous solution, whereas Solvent B was methanol with 0.1% formic acid. Elution was performed by a gradient as follows: 0 min, 2% B; 2 min, 2% B; 2.5 min, 20% B; 15 min, 80% B at the flow rate of 0.35 mL/min. The MS detection employed a QTRAP 6500 Plus with Turbo ion spray ESI source and using parameters as follows: ion spray voltage: 4500 V (positive mode) and −4500 V (negative mode); ion source temperature: 450 °C; GS1at 40 psi, GS2 at 40 psi and curtain gas at 20 psi, respectively. The neurotransmitters were identified by the MRM method with optimized MRM mother‐child ion pair information, CE, DP, and retention time. R package metaX was used to identify the differentially regulated neurotransmitters while the differentially regulated neurotransmitters between two groups were analyzed using univariate analysis methods including ratio analysis and t‐test. Neurotransmitters with ratio ≥ 1.2 or ratio ≤ 0.83 and *p* value < 0.05 were considered as differentially regulated neurotransmitters.

### Sample Preparation and LC–MS/MS Proteomic Analysis

LC‐MS/MS proteomic analysis was conducted with label‐free absolute protein quantification using data‐independent acquisition (DIA) as previously described.^[^ ^90^ ^]^ Tissue samples (5,6 mg) were transferred into a 2‐mL tube with two steel beads, and incubated in 200 µL 1×Cocktail containing sodium dodecyl sulfate (SDS) and ethylenediaminetetraacetic acid (EDTA) on ice for 5 min while 20 µL of 100nmM dithiothreitol (DTT) was supplemented. After digestion of 100 µg of proteins with 2.5 µg of trypsin in four sample volumes of 50 mM NH_4_HCO_3_ for 4 h at 37 °C, the resulted peptides were desalted using a Strata X column (Phenomenex, USA) and dried under vacuum.

For LC–MS/MS analysis, equal amounts of peptides were dissolved in Solvent A (5% ACN, pH 9.8) and analyzed on a Gemini pH stable C18 column (5 µm, 4.6 × 250 mm, Phenomenex, USA) under the control of LC‐20AB HPLC system (Shimadzu Corporation, Japan). After sample injection, the column was eluted at the flow rate of 1 mL min^−1^ by the gradient as follows: 5% Solvent B (95% ACN, pH 9.8), 10 min; 5% to 35% B, 40 min; 35% to 95% B, 1 min; 95% B, 3 min; 5% B, 10 min. The elution was monitored at the wavelength of 214 nm while the components were collected at one‐minute intervals.

Peptides were dissolved with Solvent A (2% ACN, 0.1% formic acid) and centrifuged at 20000 g for 10 min. The supernatants were collected for the analysis on a self‐packed C18 column (150 µm x 35 cm, 1.8 µm, BGI, China) under the control of UltiMate 3000 UHPLC (Thermo Fisher Scientific, USA). Following the enrichment in the trap column and desalting, the samples were separated by the gradient as follows: 5% Solvent B (98% ACN, 0.1% formic acid), 0 min; 5% B, 5 min; 120 min, 25% B; 35% B, 160 min; 80% B,170 min; 80% B, 175 min; 5% B, 180 min, at the flow rate of 500 nL min^−1^. The elution was detected by the mass spectrometer as follows: 1) For data dependent acquisition (DDA) analysis, the peptides were ionized by nanoESI and detected with DDA detection mode on Q‐Exactive HF system (Thermo Fisher Scientific, USA). The parameters were set as follows: maximal injection time (MIT), 100 ms; ion source voltage 1.9 kV; MS scan range 350–1500 m/z; MS/MS collision type, HCD; normalized collision energy (NCE), 28; dynamic exclusion duration, 30 s; start m/z for MS/MS, 100; precursors for MS/MS, charge range 2+ to 6+; intensity of top 20 precursors, over 5E4; automatic gain control (AGC), MS 3E6, MS/MS 1E5. The MS resolution was set to 120 000, while the MS/MS resolution was set to 30 000. 2) For DIA analysis, samples were ionized by nanoESI and detected with DIA detection mode on Q‐Exactive HF system. The parameters were set as follows: MIT, 50 ms; ion source voltage, 1.9 kV; MS scan range, 400–1250 m/z; MS/MS collision type, HCD; NCE (distributed mode), 22.5, 25, 27.; AGC, 1E6. MS scan range was equally divided to 45 windows for MS/MS scan. Fragment ions were detected in Orbitrap analyzer. The resolution was set to 120 000, and 30 000 for MS and MS/MS scan, respectively.

The results of the sample data were further processed by bioinformatic analysis. DDA data was analyzed through Andromeda search engine in MaxQuant and used to construct spectral library. MProphet algorithm was used to ensure analytical quality control to generate reliable quantitative results for large‐scale DIA data. GO, Cluster of Orthologous Groups of proteins (COG) and pathway functional annotation analysis were performed. The DEPs between comparison groups were identified from the quantitative results. Subsequently, the function enrichment analysis and the protein‐protein interaction analysis of the DEPs were conducted. Proteins were identified from protein databases including UniProt, reference sequence (RefSeq) of NCBI and Ensembl. Retention time calibration in the DIA data was conducted using the iRT peptides. R package MSstats was used to analyze the DEPs as described.^[^ ^91^ ^]^ DEPs screening was conducted based on the fold change >1.5 and *P* value < 0.05. GO and KEGG pathway enrichment analysis were performed on the DEPs with R.

### Cell Culture and Transfection

The highly differentiated rat pheochromocytoma cell line PC12 was acquired from the National Collection of Authenticated Cell Cultures (Shanghai, China). HT‐22 cell line was obtained from the Salk Institute for Biological Studies (San Diego, CA). Dulbecco's Modified Eagle Medium (DMEM), fetal bovine serum (FBS), horse serum (HS), and antibiotics were purchased from Thermo Fisher Scientific (Carlsbad, CA, USA). The cells were grown in DMEM supplemented with 10% heat‐inactivated HS, 5% heat‐inactivated FBS, and 1% penicillin/streptomycin, at 37 °C with a humidified atmosphere consisting of 5% CO_2_ and 95% air. For transfection, the cells were seeded at a density of 1.5 × 10^5^ cells/mL in 6‐well plates for 24 h. The pcDNA3.1‐RagA plasmid was constructed as described previously.^[^ ^7^ ^]^ The pcDNA3.1‐RagA plasmid or pcDNA3.1 vector was transfected into the cells with Effectene Transfection Reagent (QIAGEN, USA) for 24 h.

### Western Blot Analysis

The PFC tissues as a 3‐mm portion were recovered from the experimental mice and homogenized in a RIPA buffer (Sigma‐Aldrich, USA). 70 µg protein was separated by 10% SDS‐polyacrylamide gel electrophoresis (PAGE) and subsequently transferred to a polyvinylidene difluoride (PVDF) membrane (Merck Millipore, Germany). The PVDF membrane was incubated with primary antibody for RagA, phospho‐p70S6K, p70S6K, GAPDH (Cell Signaling Technology, USA), or ADORA2A (Abcam, USA), detected with horseradish peroxidase (HRP)‐conjugated anti‐rabbit secondary antibody (Sigma‐Aldrich, USA), and visualized with a select chemiluminescence detection kit (GE Healthcare, Sweden).^[^ ^92^ ^]^ The images were analyzed and the protein levels were quantified by ImageJ (http://rsb.info.nih.gov/ij/) according to the software protocols. Specifically, the original images were changed to gray images. The images were processed by subtracting image background, selecting the protein bands and setting the area, mean and integrated density. Integrated density was used for the quantification of protein levels.

### Immunostaining

The coronal sections of 40 µm thickness were obtained from the 3 mm PFC tissues. The tissue sections were incubated with antibody for NeuN, ADORA2A (Abcam, USA), or RagA (Cell Signaling Technology, USA) overnight at 4 °C and detected with Alexa Fluor 488‐conjugated goat anti‐mouse IgG secondary antibody and Alexa Fluor 594 conjugated goat anti‐rabbit IgG secondary antibody (Invitrogen, USA) for 2 h at room temperature (RT). The cell nuclei were stained with 4′‐6‐diamidino‐2‐phenylindole (DAPI) (Invitrogen, USA). The images were captured on a confocal LSM 700/800/880 fluorescence microscope (Carl Zeiss, Germany), and the fluorescence intensity was quantified by ImageJ (http://rsb.info.nih.gov/ij/) with densitometric method.^[^ ^14a^ ^]^ In practice, the color images were split to three channels. The images were processed by adjusting the threshold and setting the area, mean gray value and limit to threshold. Subsequently, the mean gray value was measured for the fluorescence quantification.

### TUNEL Assay

The broken DNAs during apoptosis were detected using the TUNEL BrightRed Apoptosis Detection Kit (Vazyme Biotech, Nanjing, China). In brief, the brain coronal frozen sections at a thickness of 8 µm were mounted on positively charged slides. The sections were fixed in 4% paraformaldehyde for 30 min, washed with PBS for three times, and incubated with Proteinase K solution for 3 min at RT. After another round of PBS washing, the sections were incubated with equilibration buffer at RT for 30 min, followed by incubation with terminal deoxynucleotidyl transferase (TdT) reaction mixture at 37 °C for 60 min. The sections were then washed with PBS and stained with DAPI. The images were captured on a confocal LSM 880 fluorescence microscope (Carl Zeiss, Germany), and the fluorescence intensity was analyzed by ImageJ (http://rsb.info.nih.gov/ij/) as previously described.^[^ ^14a^ ^]^

### Quantitative Reverse Transcriptase Polymerase Chain Reaction (qRT‐PCR)

Mouse PFC tissues of 3 mm section, highly differentiated PC12 cells, and HT‐22 cells were collected and analyzed qRT‐PCR as previously described.^[^ ^93^ ^]^ The total RNAs were extracted using TRIzol reagent (Thermo Fisher Scientific, USA) and then transcribed into the cDNAs with the RevertAid RT Reverse Transcription kit (Thermo Fisher Scientific, USA). qRT‐PCR was performed using a SYBR Green PCR kit (QIAGEN, USA and Vazyme, China). Primers for mouse genes as follows: ADORA2A‐F, 5ˊ‐CCGTGTGGATCAACAGCAACCT‐3ˊ; ADORA2A‐R, 5ˊ‐ CTCTGCGTGAGGACCAGGACAA‐3ˊ; β‐actin‐F, 5ˊ‐GGCTACAGCTTCACC‐ ACCACAG‐3ˊ; β‐actin‐R, 5ˊ‐ GGAACCGCTCGTTGCCAATAGT −3ˊ; primers for rat genes as follows: RagA‐F, 5ˊ‐GGAACCTGGTGCTGAACCTGTG‐3ˊ; RagA‐R, 5ˊ‐GGATGGCTTCCAGACACGACTG‐3ˊ; ADORA2A‐F, 5ˊ‐GGCT‐ CTTTGCTCTGTGCTGGTT‐3ˊ; ADORA2A‐R, 5ˊ‐GGTTGACGACGGAGTTG‐ CTGTG‐3ˊ; β‐actin‐F, 5ˊ‐GTATGCCTCTGGTCGTACCA‐3ˊ; β‐actin‐R, 5ˊ‐CTCTCAGCTGTGGTGGTGAA‐3ˊ. During the exponential amplification phase, the Ct values were obtained and the 2^−∆∆^Ct method was used to determine the relative expression level of target genes.

### Statistical Analysis

The data were expressed as mean±SEM for behavioral assessments in animal experiments while mean±SD for other experiments. The statistical significance was determined by unpaired *t* tests, a one‐way analysis of variance (ANOVA) followed by Dunnett's test, two‐way ANOVA followed by Šídák's multiple comparisons test with GraphPad Prism 9.5 (GraphPad Software, USA). A *p* value <0.05 was regarded as statistically significant difference. The correlations between proteins and neurotransmitters were analyzed using Spearman correlation analysis.^[^ ^94^ ^]^ A *p* value <0.05 was regarded as statistically significant correlation. The interaction network was generated from Cytoscape.

## Conflict of Interest

The authors declare no conflict of interest.

## Author Contributions

J.Z. and Y.S. conducted the experiments, Y.F. revised the manuscript, and J.R. designed the project and revised the manuscript.

## Supporting information



Supporting Information

## Data Availability

The data that support the findings of this study are available from the corresponding author upon reasonable request.
