# Supplementary material for: Brain Specific RagA Overexpression Triggers Depressive‐Like Behaviors in Mice via Activating ADORA2A Signaling Pathway
Source: Adv Sci (Weinh). 2024 Oct 7;11(45):2404188. doi: 10.1002/advs.202404188 (PMC11615787; doi:10.1002/advs.202404188)
Supplement: Supplementary file 1 — Supporting Information [file ADVS-11-2404188-s001.docx]

Supporting Information

Brain Specific RagA Overexpression Triggers Depressive-like Behaviors in Mice via Activating ADORA2A Signaling Pathway

Jia Zhao^#^, Yilu Sun^#^, Yibin Feng*, Jianhui Rong*

^#^These authors contributed equally to this work

J. Rong

School of Chinese Medicine, Li Ka Shing Faculty of Medicine, The University of Hong Kong, 3 Sassoon Road, Pokfulam, Hong Kong, China
E-mail: jrong@hku.hk

Y. Feng
School of Chinese Medicine, Li Ka Shing Faculty of Medicine, The University of Hong Kong, 3 Sassoon Road, Pokfulam, Hong Kong, China
E-mail: yfeng@hku.hk

J. Zhao, Y. Sun

School of Chinese Medicine, Li Ka Shing Faculty of Medicine, The University of Hong Kong, 3 Sassoon Road, Pokfulam, Hong Kong, China

Department of Chinese Medicine, The University of Hong Kong Shenzhen Hospital, Shenzhen, China

Keywords: depression, molecular mechanism, RagA, ADORA2A, p70S6K

**Table S1.** Correlation analysis of metabolomics and proteomics.

| Metabolites | Protein | R | p-value | R  type | p-value type |
| --- | --- | --- | --- | --- | --- |
| N-Acetylserotonin | RAMP1 | 0.894186721 | 0.000481836 | + | ** |
| Indole-3-carboxaldehyde | CG025 | 0.903030303 | 0.000880225 | + | ** |
| L-tryptophan | HECW1 | 0.890909091 | 0.001380267 | + | ** |
| gamma-aminobutyric_acid | GARE2 | -0.852824936 | 0.001711895 | - | ** |
| gamma-aminobutyric_acid | WDR83 | 0.878787879 | 0.001977059 | + | ** |
| Norepinephrine | F234A | -0.878787879 | 0.001977059 | - | ** |
| 5-hydroxytryptamine | ALKB1 | -0.866666667 | 0.002681415 | - | ** |
| Aspartate | NELFE | -0.866666667 | 0.002681415 | - | ** |
| L-tryptophan | TAF6 | -0.866666667 | 0.002681415 | - | ** |
| N-Acetylserotonin | S6A15 | -0.866666667 | 0.002681415 | - | ** |
| N-Acetylserotonin | RHG07 | -0.866666667 | 0.002681415 | - | ** |
| N-Acetylserotonin | TANC1 | 0.854545455 | 0.003504744 | + | ** |
| Indole-3-carboxaldehyde | ARP8 | -0.854545455 | 0.003504744 | - | ** |
| L-tryptophan | RIN1 | -0.854545455 | 0.003504744 | - | ** |
| Indole-3-carboxaldehyde | COX3 | 0.842424242 | 0.004459029 | + | ** |
| L-tryptophan | CTU1 | 0.842424242 | 0.004459029 | + | ** |
| N-Acetylserotonin | HOME2 | 0.842424242 | 0.004459029 | + | ** |
| Kynurenic_acid | ENOX1 | 0.803741486 | 0.005083578 | + | ** |
| N-Acetylserotonin | GRIK4 | 0.802435318 | 0.005211462 | + | ** |
| L-tryptophan | GANP | 0.83030303 | 0.005556805 | + | ** |
| N-Acetylserotonin | TM245 | 0.83030303 | 0.005556805 | + | ** |
| N-Acetylserotonin | S39A6 | 0.83030303 | 0.005556805 | + | ** |
| gamma-aminobutyric_acid | TAF6 | -0.818181818 | 0.006811133 | - | ** |
| L-glutamine | RAI1 | -0.818181818 | 0.006811133 | - | ** |
| N-Acetylserotonin | DDC | -0.818181818 | 0.006811133 | - | ** |
| N-Acetylserotonin | ZGPAT | 0.818181818 | 0.006811133 | + | ** |
| L-tryptophan | GARE2 | -0.779199761 | 0.007888893 | - | ** |
| L-tryptophan | CMTA2 | -0.806060606 | 0.008235571 | - | ** |
| Norepinephrine | CAH7 | -0.806060606 | 0.008235571 | - | ** |
| Kynurenine | UAP1 | 0.806060606 | 0.008235571 | + | ** |
| L-tryptophan | CEND | 0.806060606 | 0.008235571 | + | ** |
| Quinolinic_acid | RHG07 | 0.806060606 | 0.008235571 | + | ** |
| 5-hydroxytryptamine | TP4A2 | 0.793939394 | 0.009844136 | + | ** |
| N-Acetylserotonin | CAH7 | 0.793939394 | 0.009844136 | + | ** |
| Norepinephrine | RHG07 | 0.793939394 | 0.009844136 | + | ** |
| gamma-aminobutyric_acid | CEND | 0.781818182 | 0.011651268 | + | * |
| L-tryptophan | FHI2B | -0.781818182 | 0.011651268 | - | * |
| N-Acetylserotonin | CMTA2 | -0.781818182 | 0.011651268 | - | * |
| Norepinephrine | TM245 | -0.781818182 | 0.011651268 | - | * |
| Kynurenine | GRIK4 | -0.753802874 | 0.011794786 | - | * |
| Indole-3-carboxaldehyde | GANP | 0.76969697 | 0.013671782 | + | * |
| L-tyrosine | PPR37 | -0.76969697 | 0.013671782 | - | * |
| N-Acetylserotonin | PP6R2 | 0.76969697 | 0.013671782 | + | * |
| N-Acetylserotonin | CRHBP | 0.741644763 | 0.014075178 | + | * |
| 5-hydroxytryptamine | MAST1 | -0.757575758 | 0.015920829 | - | * |
| Acetylcholine | SCO1 | 0.757575758 | 0.015920829 | + | * |
| Aspartate | CG025 | 0.757575758 | 0.015920829 | + | * |
| gamma-aminobutyric_acid | COX3 | 0.757575758 | 0.015920829 | + | * |
| L-5-hydroxytryptophan | COX3 | 0.757575758 | 0.015920829 | + | * |
| L-tryptophan | FRS2 | 0.757575758 | 0.015920829 | + | * |
| L-tryptophan | OPTN | 0.757575758 | 0.015920829 | + | * |
| N-Acetylserotonin | CTNA1 | -0.757575758 | 0.015920829 | - | * |
| N-Acetylserotonin | AT2C1 | 0.757575758 | 0.015920829 | + | * |
| N-Acetylserotonin | FHI2B | -0.757575758 | 0.015920829 | - | * |
| N-Acetylserotonin | FRS2 | 0.757575758 | 0.015920829 | + | * |
| N-Acetylserotonin | KLD8B | 0.757575758 | 0.015920829 | + | * |
| 5-hydroxytryptamine | NMNA3 | 0.730116312 | 0.016506245 | + | * |
| 5-hydroxytryptamine | EFGM | 0.745454545 | 0.018413841 | + | * |
| Aspartate | RAI1 | -0.745454545 | 0.018413841 | - | * |
| Aspartate | GANP | 0.745454545 | 0.018413841 | + | * |
| Epinephrine | F234A | -0.745454545 | 0.018413841 | - | * |
| Glutathione | TAF6 | -0.745454545 | 0.018413841 | - | * |
| L-5-hydroxytryptophan | ARP8 | -0.745454545 | 0.018413841 | - | * |
| L-tryptophan | IPO13 | -0.745454545 | 0.018413841 | - | * |
| 3-Hydroxyanthranilic_acid | S6A15 | -0.733333333 | 0.021166481 | - | * |
| 5-hydroxytryptamine | NOP16 | 0.733333333 | 0.021166481 | + | * |
| Kynurenic_acid | DLGP1 | 0.733333333 | 0.021166481 | + | * |
| L-tyrosine | CEND | 0.733333333 | 0.021166481 | + | * |
| N-Acetylserotonin | ANO3 | -0.733333333 | 0.021166481 | - | * |
| N-Acetylserotonin | DCAKD | 0.733333333 | 0.021166481 | + | * |
| N-Acetylserotonin | DEN6B | 0.733333333 | 0.021166481 | + | * |
| Quinolinic_acid | FHI2B | 0.733333333 | 0.021166481 | + | * |
| Acetylcholine | ENOX1 | 0.705574587 | 0.022625593 | + | * |
| 3-Hydroxyanthranilic_acid | A16A1 | 0.721212121 | 0.024194588 | + | * |
| 5-hydroxytryptamine | MED22 | -0.721212121 | 0.024194588 | - | * |
| Dopammine | WDR83 | 0.721212121 | 0.024194588 | + | * |
| gamma-aminobutyric_acid | NTAQ1 | 0.721212121 | 0.024194588 | + | * |
| Glutathione | CTU1 | 0.721212121 | 0.024194588 | + | * |
| Kynurenic_acid | MED22 | -0.721212121 | 0.024194588 | - | * |
| L-glutamine | PPOX | 0.69301232 | 0.026292878 | + | * |
| 3-Hydroxyanthranilic_acid | ANO3 | -0.709090909 | 0.027514119 | - | * |
| 5-hydroxyindole-3-acetic_acid | CTU1 | -0.709090909 | 0.027514119 | - | * |
| Acetylcholine | FHI2B | -0.709090909 | 0.027514119 | - | * |
| Acetylcholine | RIN1 | -0.709090909 | 0.027514119 | - | * |
| L-tryptophan | CFAB | -0.709090909 | 0.027514119 | - | * |
| N-Acetylserotonin | NELFE | -0.709090909 | 0.027514119 | - | * |
| Indole-3-carboxaldehyde | LMTK3 | 0.709090909 | 0.027514119 | + | * |
| Indole-3-carboxaldehyde | CTU1 | 0.709090909 | 0.027514119 | + | * |
| L-tryptophan | LMTK3 | 0.709090909 | 0.027514119 | + | * |
| L-tryptophan | PP6R2 | 0.709090909 | 0.027514119 | + | * |
| 5-hydroxytryptamine | FSTL1 | 0.681032862 | 0.030150884 | + | * |
| L-tryptophan | CSMD1 | 0.681032862 | 0.030150884 | + | * |
| L-tryptophan | NMNA3 | 0.681032862 | 0.030150884 | + | * |
| N-Acetylserotonin | FRPD1 | -0.680854209 | 0.030211171 | - | * |
| 5-hydroxytryptamine | NCOA5 | 0.696969697 | 0.031141095 | + | * |
| Alpha-ketoglutaric_acid | IFT25 | -0.696969697 | 0.031141095 | - | * |
| Aspartate | SLIK2 | 0.696969697 | 0.031141095 | + | * |
| Dopammine | RIN1 | -0.696969697 | 0.031141095 | - | * |
| Dopammine | CTU1 | 0.696969697 | 0.031141095 | + | * |
| Glutamic_acid | NELFE | -0.696969697 | 0.031141095 | - | * |
| Glutathione | WDR83 | 0.696969697 | 0.031141095 | + | * |
| Kynurenic_acid | WDR83 | 0.696969697 | 0.031141095 | + | * |
| Kynurenine | B4GT3 | -0.696969697 | 0.031141095 | - | * |
| L-arginine | COX3 | 0.696969697 | 0.031141095 | + | * |
| L-histidine | NELFE | -0.696969697 | 0.031141095 | - | * |
| L-tryptophan | DDC | -0.696969697 | 0.031141095 | - | * |
| L-tryptophan | S6A15 | -0.696969697 | 0.031141095 | - | * |
| L-tryptophan | DCAKD | 0.696969697 | 0.031141095 | + | * |
| L-tryptophan | ZGPAT | 0.696969697 | 0.031141095 | + | * |
| L-tryptophan | NCOA5 | 0.696969697 | 0.031141095 | + | * |
| Metanephrine | FMT | 0.696969697 | 0.031141095 | + | * |
| N-Acetylserotonin | KHNYN | -0.696969697 | 0.031141095 | - | * |
| Quinolinic_acid | PP6R2 | -0.696969697 | 0.031141095 | - | * |
| L-tryptophan | RAMP1 | 0.675329831 | 0.032116143 | + | * |
| Dopammine | GARE2 | -0.668762 | 0.034485189 | - | * |
| L-tryptophan | CRHBP | 0.668696098 | 0.034509542 | + | * |
| 3-Hydroxyanthranilic_acid | UAP1 | -0.684848485 | 0.035091538 | - | * |
| 5-hydroxytryptamine | KHNYN | -0.684848485 | 0.035091538 | - | * |
| Histamine | FMT | -0.684848485 | 0.035091538 | - | * |
| L-Dopa | F234A | -0.684848485 | 0.035091538 | - | * |
| N-Acetylserotonin | RIN1 | -0.684848485 | 0.035091538 | - | * |
| Quinolinic_acid | HECW1 | -0.684848485 | 0.035091538 | - | * |
| Vanillmandelic_acid | COX3 | -0.684848485 | 0.035091538 | - | * |
| Aspartate | COX3 | 0.684848485 | 0.035091538 | + | * |
| Glutamic_acid | CG025 | 0.684848485 | 0.035091538 | + | * |
| Kynurenic_acid | TAOK3 | 0.684848485 | 0.035091538 | + | * |
| L-5-hydroxytryptophan | CG025 | 0.684848485 | 0.035091538 | + | * |
| L-glutamine | SI1L1 | 0.684848485 | 0.035091538 | + | * |
| L-tryptophan | DCA11 | 0.684848485 | 0.035091538 | + | * |
| L-tryptophan | DLGP1 | 0.684848485 | 0.035091538 | + | * |
| L-tryptophan | DEN6B | 0.684848485 | 0.035091538 | + | * |
| Melatonine | GGYF2 | 0.684848485 | 0.035091538 | + | * |
| 5-hydroxytryptamine | CK5P3 | 0.656491137 | 0.039223265 | + | * |
| Tryptamine | FSTL1 | 0.656491137 | 0.039223265 | + | * |
| Acetylcholine | CTU1 | 0.672727273 | 0.039381409 | + | * |
| gamma-aminobutyric_acid | CTU1 | 0.672727273 | 0.039381409 | + | * |
| Glutathione | HECW1 | 0.672727273 | 0.039381409 | + | * |
| Kynurenine | HOME2 | -0.672727273 | 0.039381409 | - | * |
| L-tryptophan | TANC1 | 0.672727273 | 0.039381409 | + | * |
| L-tryptophan | KHNYN | -0.672727273 | 0.039381409 | - | * |
| L-tyrosine | GGYF2 | -0.672727273 | 0.039381409 | - | * |
| N-Acetylserotonin | ERLEC | -0.672727273 | 0.039381409 | - | * |
| N-Acetylserotonin | B4GT3 | 0.672727273 | 0.039381409 | + | * |
| Quinolinic_acid | TM245 | -0.672727273 | 0.039381409 | - | * |
| Quinolinic_acid | DDC | 0.672727273 | 0.039381409 | + | * |
| Quinolinic_acid | HOME2 | -0.672727273 | 0.039381409 | - | * |
| Quinolinic_acid | GRIK4 | -0.650458932 | 0.041705451 | - | * |
| 5-hydroxytryptamine | CFAB | -0.660606061 | 0.044026553 | - | * |
| 5-hydroxytryptamine | DPOE4 | 0.660606061 | 0.044026553 | + | * |
| Acetylcholine | HECW1 | 0.660606061 | 0.044026553 | + | * |
| Indole-3-carboxaldehyde | RER1 | 0.660606061 | 0.044026553 | + | * |
| Kynurenine | DDC | 0.660606061 | 0.044026553 | + | * |
| L-glutamine | SLIK2 | 0.660606061 | 0.044026553 | + | * |
| L-tryptophan | SCO1 | 0.660606061 | 0.044026553 | + | * |
| N-Acetylserotonin | F234A | 0.660606061 | 0.044026553 | + | * |
| Indole-3-carboxaldehyde | FSTL1 | 0.644220275 | 0.044381366 | + | * |
| L-tryptophan | CK5P3 | 0.644220275 | 0.044381366 | + | * |
| N-Acetylserotonin | CK5P3 | 0.644220275 | 0.044381366 | + | * |
| Aspartate | RIN1 | -0.648484848 | 0.049042632 | - | * |
| L-tryptophan | MAST1 | -0.648484848 | 0.049042632 | - | * |
| 3-Hydroxyanthranilic_acid | ZGPAT | 0.648484848 | 0.049042632 | + | * |
| Aspartate | AT2C1 | 0.648484848 | 0.049042632 | + | * |
| Dopammine | TAOK3 | 0.648484848 | 0.049042632 | + | * |
| gamma-aminobutyric_acid | OPTN | 0.648484848 | 0.049042632 | + | * |
| gamma-aminobutyric_acid | HECW1 | 0.648484848 | 0.049042632 | + | * |
| gamma-aminobutyric_acid | DLGP1 | 0.648484848 | 0.049042632 | + | * |
| L-tryptophan | RN121 | 0.648484848 | 0.049042632 | + | * |
| Metanephrine | LYN | 0.648484848 | 0.049042632 | + | * |
| Metanephrine | CMC2 | 0.648484848 | 0.049042632 | + | * |
| N-Acetylserotonin | DPOE4 | 0.648484848 | 0.049042632 | + | * |
| Tryptophol | SCO1 | 0.648484848 | 0.049042632 | + | * |
| Kynurenic_acid | GARE2 | -0.631949413 | 0.049975087 | - | * |
| Dopammine | CSMD1 | 0.631949413 | 0.049975087 | + | * |
| L-tryptophan | FSTL1 | 0.631949413 | 0.049975087 | + | * |
| L-tryptophan | ENOX1 | 0.631949413 | 0.049975087 | + | * |
| N-Acetylserotonin | NMNA3 | 0.631949413 | 0.049975087 | + | * |
| Tryptophol | ENOX1 | 0.631949413 | 0.049975087 | + | * |

**Table S2.** Antibody information.

| Antibodies | Source | Identifier | Dilution |
| --- | --- | --- | --- |
| Anti-RagA | Cell Signaling Technology | Cat#4357S | 1:1000/1:100 |
| Anti-p-p70S6 | Cell Signaling Technology | Cat#9205S | 1:1000 |
| Anti-p70S6 | Cell Signaling Technology | Cat#9202S | 1:1000 |
| Anti-GAPDH | Cell Signaling Technology | Cat#2118l | 1:1000 |
| Anti-NeuN | Abcam | Cat#ab104224 | 1:200 |
| Anti-ADORA2A | Abcam | Cat#ab3461 | 1:1000/1:150 |
| Alexa Fluor 594 goat anti-rabbit | Invitrogen | Cat#A11012 | 1:500 |
| Alexa Fluor 488 goat anti-mouse | Invitrogen | Cat#A11001 | 1:500 |
| Anti-rabbit | Sigma Aldrich | Cat#A0545 | 1:500 |
